# Wheat Amylase Trypsin Inhibitors Aggravate Intestinal Inflammation Associated with Celiac Disease Mediated by Gliadin in BALB/c Mice

**DOI:** 10.3390/foods11111559

**Published:** 2022-05-25

**Authors:** Tian Yu, Shuai Hu, Fangfang Min, Jingjing Li, Yunpeng Shen, Juanli Yuan, Jinyan Gao, Yong Wu, Hongbing Chen

**Affiliations:** 1 State Key Laboratory of Food Science and Technology, Nanchang University, Nanchang 330047, China; yutian@email.ncu.edu.cn (T.Y.); 352313320029@email.ncu.edu.cn (F.M.); lijingjing77@email.ncu.edu.cn (J.L.); syp@email.ncu.edu.cn (Y.S.); yuanjuanli@ncu.edu.cn (J.Y.); chenhongbing@ncu.edu.cn (H.C.); 2 Sino-German Joint Research Institute, Nanchang University, Nanchang 330047, China; 3 School of Food Science and Technology, Nanchang University, Nanchang 330031, China; gaojy2013@ncu.edu.cn; 4 Institute of Agricultural Products Processing, Jiangxi Academy of Agricultural Sciences, Nanchang 330200, China; shu@email.ncu.edu.cn; 5 School of Pharmaceutical Science, Nanchang University, Nanchang 330006, China

**Keywords:** wheat amylase trypsin inhibitors, celiac disease, gliadin, intestinal inflammation, Th1/Th2

## Abstract

Celiac disease (CD) is an autoimmune intestinal disorder caused by the ingestion of gluten in people who carry the susceptible gene. In current celiac disease research, wheat gluten is often the main target of attention, neglecting the role played by non-gluten proteins. This study aimed to describe the effects of wheat amylase trypsin inhibitors (ATI, non-gluten proteins) and gliadin in BALB/c mice while exploring the further role of relevant adjuvants (cholera toxin, polyinosinic: polycytidylic acid and dextran sulfate sodium) intervention. An ex vivo splenocyte and intestinal tissue were collected for analysis of the inflammatory profile. The consumption of gliadin and ATI caused intestinal inflammation in mice. Moreover, the histopathology staining of four intestinal sections (duodenum, jejunum, terminal ileum, and middle colon) indicated that adjuvants, especially polyinosinic: polycytidylic acid, enhanced the villi damage and crypt hyperplasia in co-stimulation with ATI and gliadin murine model. Immunohistochemical results showed that tissue transglutaminase and IL-15 expression were significantly increased in the jejunal tissue of mice treated with ATI and gliadin. Similarly, the expression of inflammatory factors (TNF-α, IL-1β, IL-4, IL-13) and Th1/Th2 balance also showed that the inflammation response was significantly increased after co-stimulation with ATI and gliadin. This study provided new evidence for the role of wheat amylase trypsin inhibitors in the pathogenesis of celiac disease.

## 1. Introduction

As one of the world’s top three food crops [1], wheat is cultivated world-wide due to its unique adaptability and high yield [2]. Wheat is especially highly appreciated because of its ability to make dough from its flour for preparing baked food products with characteristic taste, smell and high nutritional value [3]. Despite its wide appreciation, some people experience negative health effects after consumption of wheat-based products, including several forms of wheat allergy, wheat sensitivity, and wheat intolerance including celiac disease and dermatitis herpetiformis [4,5,6]. These effects relate to a variety of wheat proteins.

Celiac disease (CD) is a chronic inflammation of the intestine, often presenting as villi damage and crypt hyperplasia, which can cause malabsorption of nutrients due to an autoimmune response caused by consumption of wheat gluten [7], especially its gliadin component. When the gluten protein reaches the lamina propria of intestine, the glutamine residues in the immunodominant peptides (such as p57–68 and 33-mer) of gliadin are deamidated into glutamate by the tissue transglutaminase (tTG) [8]. Meanwhile, the deamidation of glutamine is conducive to mediate the formation of immunostimulatory epitopes and produce strong binding affinity with Human Leukocyte Antigen-DQ2/DQ8 (HLA-DQ2/DQ8) class II compounds attached to antigen presenting cells (APC) [9]. The deamidated gluten peptides increase the presentation to cells, which stimulates the immune system [10]. Moreover, the activated T cells secrete Th1-type cytokines, which lead to infiltration of intestinal epithelial inflammatory cells and formation of mucosa [11,12].

The innate immune system responds early to multiple microbial and chemical irritants, which are essential to successfully elicit adaptive immunity [13]. Wheat amylase trypsin inhibitors (ATI), non-gluten-related protein components of wheat flour, are often identified as triggers for several wheat allergies and non-celiac gluten sensitivity and activators of innate immunity [14,15,16,17]. Actually, ATI may also aggravate inflammatory bowel disease (IBD) and non-intestinal inflammation and related immune responses [18]. Recent studies in cell and animal (mice) model systems reported that, next to gluten, ATI can also be involved in CD [13,19,20,21]. ATI may be an important inducer of intestinal innate immune responses in CD via the Toll-like receptor 4 (TLR4)-MD2-CD14 pathway [13,22] and induce intestinal barrier dysfunction and immune activation [20].

When sensitive people consume gluten-containing foods, they will inevitably ingest ATI concomitantly, which then may up-regulate the innate immune system and aggravate symptoms [14,23]. Based on the celiac toxic effect of gliadin and the innate immune activation of ATI, we speculated that ATI could exacerbate the role of gliadin in the pathogenesis of celiac disease. Furthermore, we tried to use cholera toxin (CT), polyinosinic: polycytidylic acid (Poly:IC) and dextran sulfate sodium (DSS) to further aggravate the intestinal inflammation in mice. CT is used as an adjuvant as its non-toxic subunits are able to associate with intestinal epithelial cell receptors, thereby increasing the absorption of allergens and aggravating the symptoms of reactions [24]. Poly:IC and DSS are used to induce low levels of chronic jejunum/ileitis and colitis [20], respectively.

In current celiac disease research, wheat gluten is often the main target of attention, neglecting the role played by non-gluten proteins. We aimed to describe the effects of ATI and gliadin (Gli) in BALB/c mice while exploring the further role of relevant adjuvants (CT, Poly:IC and DSS) intervention. In this study, the effects of gliadin, ATIs and related adjuvants on the intestinal inflammation of BALB/c mice were systematically assessed by the clinical scores, temperature, weight, histological sections of intestine, immunohistochemistry analysis of tissue transglutaminase and IL-15, the differentiation of Th1/Th2 and the expression of inflammatory factors secreted by splenocytes.

## 2. Materials and Methods

### 2.1. Materials and Reagents

The extraction and purification of ATI were prepared as described by Zevallos, V. F [20]. Briefly, high-gluten wheat flour (*Triticum aestivum* L.) was extracted by using ammonium bicarbonate buffer, the supernatant was fractionally precipitated by using ammonium sulfate, and then the mixture was dialyzed, sterile filtrated, lyophilized and further purified by FPLC. Gliadin from wheat, bovine serum albumin (BSA), Poly:IC and DSS (MW: 40,000 Da) were purchased from Sigma-Aldrich (Sigma-Aldrich, St. Louis, MO, USA). Sodium salt of Caboxy Methyl Cellulose (CMC) with food grade was obtained from Jiangsu Tailida New Materials CO., Ltd. (Jiangsu Tailida, Nantong, China). Cholera Toxin (CT, B subunit) and tissue lysate were acquired from Absin (Absin, Shanghai, China). Mouse IL-1β, TNF-α, IL-4 and IL-13 enzyme-linked immunosorbent assay kits were purchased from Luminex (Univ-bio, Shanghai, China). APC anti-mouse IL-4, PE/Cyanine 7 anti-mouse CD4, FITC anti-mouse INF-γ and Fixable Viability Stain 510 were procured from BD Pharmingen (BD Biosciences, Piscataway, NJ, USA). Other analytical grade reagents were purchased from Xilong Scientific (Xilong Scientific, Guangdong, China).

### 2.2. Animals and Diets

The specific pathogen-free (SPF) 4–6-week-old BALB/c mice (20 ± 2.0 g) were obtained from Hunan Slake Jingda Laboratory Animal Co., Ltd. (Hunan Slake Jingda, Changsha, China) (License No. SCXK (Hunan) 2016-0002) and the mice utilized in this research were taken care of in accordance with the Guide to Animal Experimentation at Science and Technology Laboratory Animal Research center of Jiangxi University of Traditional Chinese Medicine, Nanchang, China (License No. SYXK (Jiangxi) 2017-0004). All experimental procedures were examined and ratified by the Experimental Animal Ethics Committee of Jiangxi University of Traditional Chinese Medicine (No. JZLLSC2019-0094; date of approval: 3 November 2019). The mice were maintained on a gluten-free diet (based on AIN-93 standard, Trophic Animal Feed High-Tech Co., Ltd., Nantong, China) with the specified carbohydrate content and casein as the major source of protein and bred for at least two generations. The female mice of the second generation and above were more susceptible to gluten and were utilized as experimental animals. The room temperature and humidity were maintained under conditions of 21 °C–25 °C and 55%–65%, respectively, with 12 h dark and light cycle.

### 2.3. Animal Experiment

**Figure 1:**
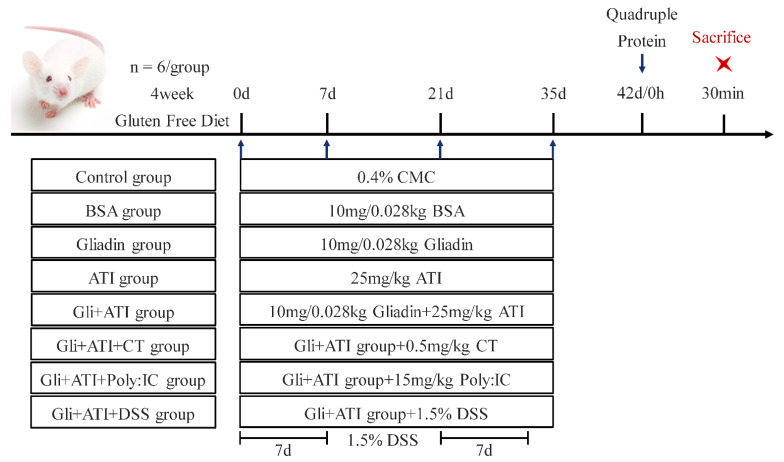
Timeline of experimental protocol.

Female mice were allowed to drink and eat freely for a week to adapt to the new environment, and were then randomly assigned into eight groups of six each, named the Control group, BSA group, Gliadin group, ATI group, Gli+ATI group, Gli+ATI+CT group, Gli+ATI+Poly:IC group and Gli+ATI+DSS group, respectively. Here, 0.4% CMC was used as a solvent to promote the dissolution of gliadin. All mice would be challenged by gavage on days 0, 7, 21 and 35, which were given BSA or gliadin with the same amount of protein (10 mg/0.028 kg BSA/gliadin + 25 mg/kg ATIs or 0.5 mg/kg CT or 15 mg/kg Poly: IC or 1.5% DSS) as described by Vijaykrishnaraj [25] and Zevallos [20]. The mice were stimulated on day 42 using quadruple doses of protein (Figure 1). The mice were given Poly:IC by intraperitoneal injection and DSS by drinking DSS-dissolving. The mice in the negative control group were received 0.4% CMC (0.2 mL/10 g mouse) by intragastric gavage (sham treatment) and the mice of all groups (expect Gli+ATI+Poly:IC group, Poly:IC’s solvent is physiological saline) were given physiological saline (0.1 mL/10 g mouse) by intraperitoneal injection. After 30 min of sensitization on day 42, the mice were scored for clinical symptoms, and their body temperature and weight were recorded. After being anaesthetized by isoflurane, the mice were euthanized by dislocation. The whole intestine and spleen of mice were collected, respectively.

### 2.4. Histological Observation

The intestinal tissues (colon, ileum, jejunum and duodenum) of mice, which were fixed in 4% paraformaldehyde solution and embedded in paraffin, were sectioned and stained by using hematoxylin and eosin. After the sections had been dehydrated and sealed with neutral gum, they were viewed and documented using a light microscope (upright optical microscope Nikon Eclipse E100 and imagine system Nikon DS-U3, Nikon Co., Tokyo, Japan).

### 2.5. Immunohistochemistry of Jejunum Tissue Sections

The sections of paraffin-embedded jejunum tissues were used for immunohistochemistry (IHC) of tissue transglutaminase (tTG) and IL-15, which were incubated with anti-mouse tTG, anti-mouse IL-15 and secondary antibody (HRP labeled) according to the manufacturer’s protocols. The stained parts were observed and photographed under an optical microscope (200× magnification). The IHC data were quantified using Image J software. Different parts of the same tissue would be analyzed.

### 2.6. Splenocyte Culture In Vitro and Cytokine Analysis

Mice spleens were ground in RPMI 1640 medium under aseptic conditions. The ground spleens were lysed 5 min by erythrocyte lysate, then the supernatant was abandoned by centrifugation at 300 g 5 min. The cells in precipitation were resuspended by RPMI 1640 twice and transferred to complete medium (RPMI-1640 with 10% fetal bovine serum). All operations were performed on ice to ensure a low temperature environment. The cells were seeded at a density of 5 × 10^6^/mL on 24-well cell culture plate. After that, cells were stimulated by the peptides (1 mg/mL) of BSA/gliadin/ATIs digestion obtained by the method of Martin Wickham et al. [26], respectively. After 72 h of incubation at 37 °C with 5% CO_2_, supernatants of cells were gathered for storage at −80 °C and the levels of IL-1β, IL-4, IL-13 and TNF-α in splenocytes were analyzed.

### 2.7. Th1 and Th2 Cell Subsets of Splenocytes

According to the difference in characteristic cytokines secreted by cell subpopulations, the expression of Th1 and Th2 cell subsets was measured indirectly by the intracellular production levels of IFN-γ and IL-4. The splenocytes whose cell concentration had been adjusted were stimulated 15 h by cell stimulation cocktail, and then, the splenocytes were divided into control group, sample group, set-up group, FMO group and compensation group. Afterward, a 100 µL suspension of splenic single cells was obtained and washed with ice-cold PBS without protein components. Fixable Viability Stain 510 was put into the corresponding group and hatched in darkness at 4 °C 30 min. Subsequently, the splenocytes were washed with PBS containing 2% fetal bovine serum, PE/Cyanine 7 anti-mouse CD4 was added in the corresponding group, and this was incubated in the dark. After the addition of fixative and rupture agents in the cell suspension, the cells were collected. APC anti-mouse IL-4 and FITC anti-mouse INF-γ antibodies were added into resuspended cells and incubated in dark. Later, the individual splenocytes were resuspended in 400 μL ice-cold PBS and went through the 100 meshes nylon mesh. Finally, Th1 and Th2 cell subsets were counted using flow cytometry (Becton, Dickinson and Company, New York, NY, USA).

### 2.8. Statistical Analysis

All experimental values were represented as mean ± standard deviation (SD), resulting from at least three independent data. Immunohistochemistry related images were evaluated using the IHC toolbox plugin of Image J software. The average optical density (AOD) was used to calculate the positive area of immunohistochemistry. The data of flow cytometry was performed by using the BD FACSuite and FlowJo V10 software. GraphPad Prism 8 and PowerPoint 2016 software were utilized to draw the charts. The data were statistically analyzed using one-way ANOVA analysis in IBM SPSS Statistics 24 software with Tukey’s HSD test and independent sample t-test. Probability values of *p* < 0.05 were considered significantly different.

## 3. Results

### 3.1. Weight, Water Consumption, Clinical Symptoms and Body Temperature of Mice

After the second-generation female mice were given four weeks of the gluten-free diet, the mice were exposed to a gliadin-containing diet which were supplemented with ATI and related adjuvants (Figure 1). 

**Figure 2:**
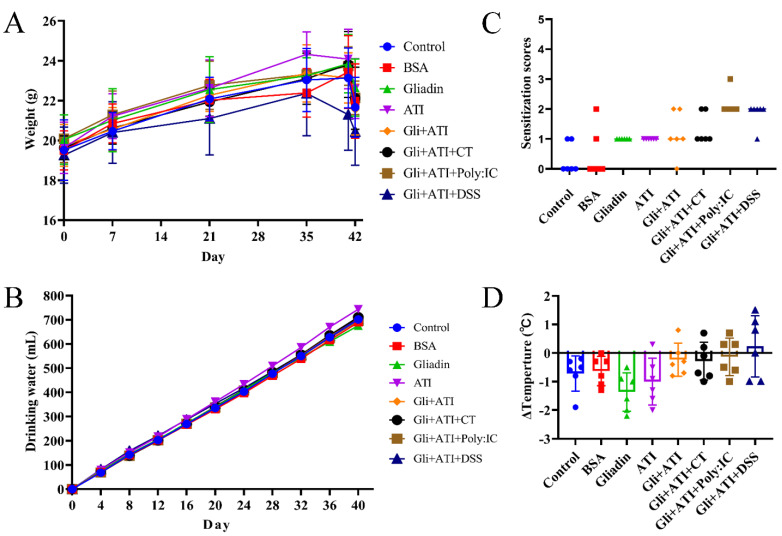
Weight, water consumption, clinical symptoms and body temperature of mice. (**A**) Body weight of mice. (**B**) Water consumption of mice. (**C**) Clinical signs scores of mice. (**D**) Changes of mice body temperature. (ΔTemperature = body temperature of mice after stimulation - body temperature of mice before stimulation). Footnote: Clinical symptoms score: (0) No symptoms; (1) Scratching nose and mouth; (2) Swelling around the eyes and mouth, diarrhea, reduced activity or walking in place, higher breathing rate; (3) Shortness of breath, wheezing, blue rash around the mouth and tail; (4) Loss of consciousness, tremors or cramps; (5) Death by shock.

The body weight of mice was recorded each time when they were orally challenged (Figure 2A). No significant change in the body weights of the animals was observed between groups in the first week. After that, only the mice in the Gli+ATI+DSS group showed a trend of weight loss compared to the other groups. We measured the daily water intake of mice in order to exclude the interference of DSS on their water intake and found no significant difference in water consumption (Figure 2B). Moreover, our results showed that mice in both the Gliadin and ATI groups exhibited scratching, while two mice in the Gli+ATI group exhibited diarrhea (Figure 2C). The symptoms of the mice were further aggravated by the addition of Poly:IC or DSS. The Gli+ATI+Ploy:IC group mice had the most severe symptoms, with five mice exhibiting diarrhea and one mouse exhibiting shortness of breath. The changes in the mouse rectal temperature after quadruple protein challenge are shown in Figure 2D. The mice in the Gliadin group and ATI group dropped to the lowest body temperature after stimulation. In contrast, co-stimulation with gliadin and ATI resulted in an increase in the mice’s body temperature compared with Control group.

### 3.2. Histology of Intestine Tissue Section

**Figure 3:**
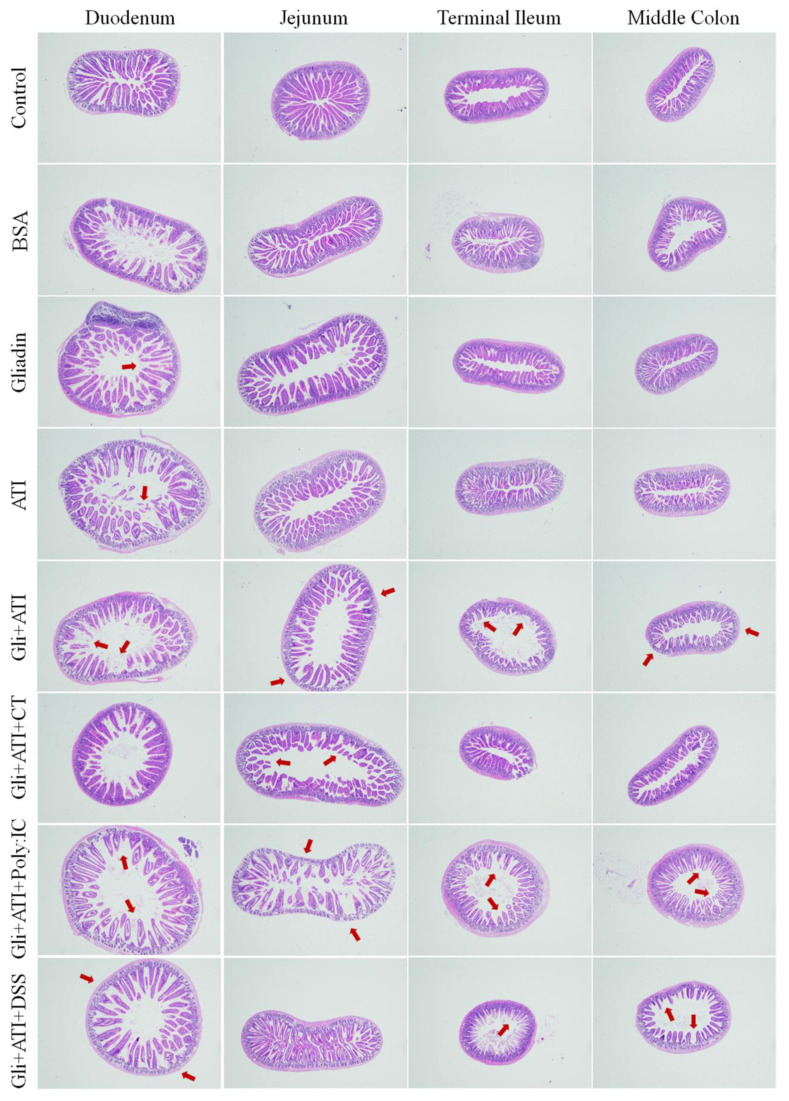
Intestine histological sections of terminal ileum, middle colon, jejunum and duodenum stained with hematoxylin and eosin stain. The images are the fields of view at 40× lens.

The pathological features and changes in the histological sections of intestine (including duodenum, jejunum, terminal ileum and middle colon) are shown in Figure 3 and Supplementary Figure S1. The mice in the Gliadin and ATI groups demonstrated mild atrophy and infiltration at four gut sites compared to the control group. In duodenum, terminal ileum and middle colon sections of mice, the Gli+ATI group, Gli+ATI+Poly:IC group and Gli+ATI+DSS group showed severe villous atrophy. In addition, the Gli+ATI group, the Gli+ATI+CT group and the Gli+ATI+Poly:IC group exhibited severe crypt hyperplasia and infiltration in the jejunum tissue sections of mice. Taken together, the intestinal villous atrophy, crypt hyperplasia and infiltration in mice would be exacerbated by the co-stimulation of gliadin and ATI, with the Gli+ATI+Poly:IC group showing the most severe intestinal damage in the four tissue sections of the intestine.

### 3.3. Immunohistochemistry of The Sections of Jejunum Tissue

**Figure 4:**
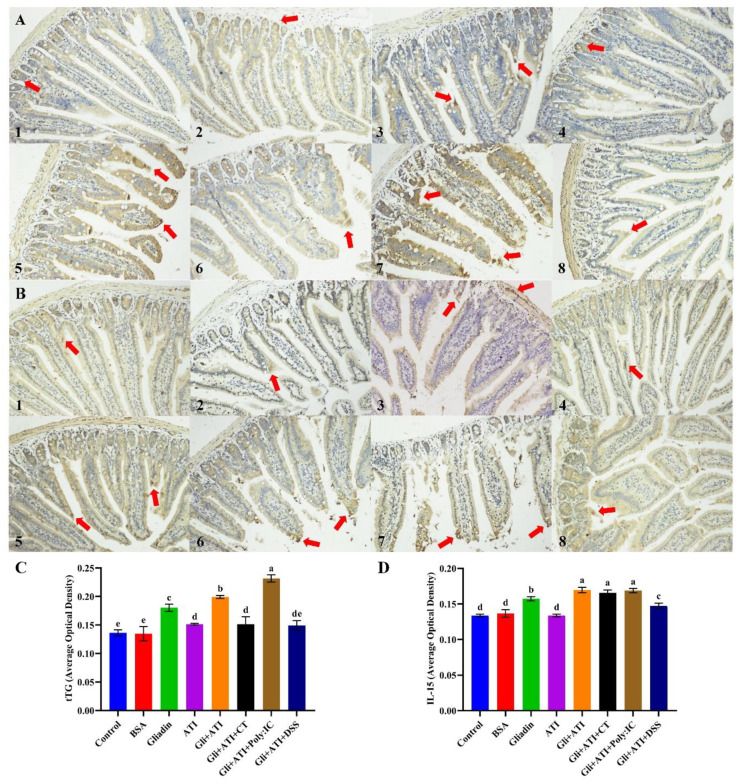
Immunohistochemistry analysis of tissue transglutaminase (**A**) and IL-15 (**B**) in jejunum tissue sections: (1) Control group; (2) BSA group; (3) Gliadin group; (4) ATI group; (5) Gli+ATI group; (6) Gli+ATI+CT group; (7) Gli+ATI+Poly:IC group; (8) Gli+ATI+DSS group. (**A**,**B**) are the fields of view at 200× lens. The average optical density of tissue transglutaminase (**C**) and IL-15 (**D**). Different parts of the same tissue would be analyzed in order to obtain reliable results. Different letters represent significant differences (*p* < 0.05) between groups.

The immunohistochemistry analyses of tissue transglutaminase and IL-15 in the jejunum tissue sections are shown in Figure 4. As shown in Figure 4A,C, gliadin could significantly increase the expression of tTG in mice jejunal tissue compared with control group (*p* < 0.05). Co-stimulation with gliadin and ATI further promoted the expression of tTG (*p* < 0.05). In addition, the adding of Poly:IC caused the expression of tTG in mice jejunum to become the highest. Like the results of tTG, gliadin also significantly increased the expression of IL-15 in mice jejunal tissue, as shown in Figure 4B,D (*p* < 0.05). The co-stimulation of gliadin and ATI also exacerbated the expression of IL-15 in mice jejunum compared with the Gliadin group (*p* < 0.05). By contrast, neither CT nor Poly:IC further enhanced the levels of IL-15 in jejunal tissues of mice in the Gli+ATI group. Overall, the co-stimulation of gliadin and ATI significantly increased the levels of tTG and IL-15 in mice jejunum tissue, while the extra addition of Poly:IC further aggravated tTG expression in mice jejunum.

### 3.4. Differentiation and Homeostasis of Th1/Th2

**Figure 5:**
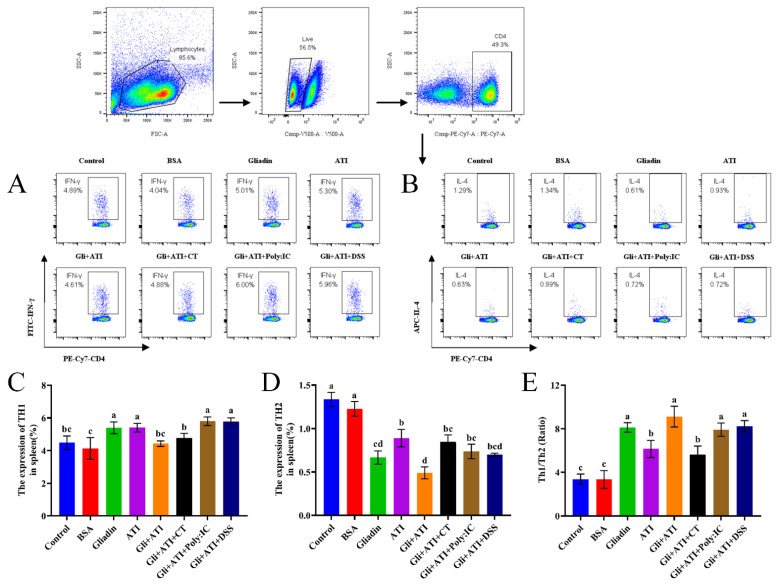
The homeostasis of differentiation of Th1 and Th2 subpopulations in splenocytes of mouse using flow cytometry. (**A**,**B**) T-helper type 1 (Th1) and T-helper type 2 (Th2) cell subpopulations. (**C**,**D**) The expression percentage of Th1 and Th2 subpopulations. (**E**) The ratios of Th1/Th2 cells. Different letters represent significant differences (*p* < 0.05) between groups.

Being one of the largest immune organs in the body, the spleen often has a crucial role in regulating immune responses and thus we performed related assays on the spleens of mice. The expression of Th1/Th2 in mice splenocytes are shown in Figure 5. As shown in Figure 5A,C, the expression of Th1 cells was increased significantly in the Gliadin group, ATI group, Gli+ATI+Poly:IC group and Gli+ATI+DSS group compared with that in control group (4.89%). No significant differences were found between the Gli+ATI group, Gli+ATI+CT group and the control group. As shown in Figure 5B,D, Th2 expression was significantly reduced in all experimental groups compared with control group (1.29%). The lowest expression levels were observed in the Gliadin group and Gli+ATI group (0.61%, 0.63%, respectively). In addition, we further compared the ratios of Th1 and Th2 cells in murine spleens (Figure 5E). All of Gliadin group, Gli+ATI group, Gli+ATI+Poly:IC group and Gli+ATI+DSS group showed a higher ratio of Th1/Th2. Taken together, co-stimulation of Gliadin and ATI could severely disrupt the balance between Th1 and Th2 cells and develop the direction towards immunity of Th1.

### 3.5. The Levels of Splenocyte Cytokines

**Figure 6:**
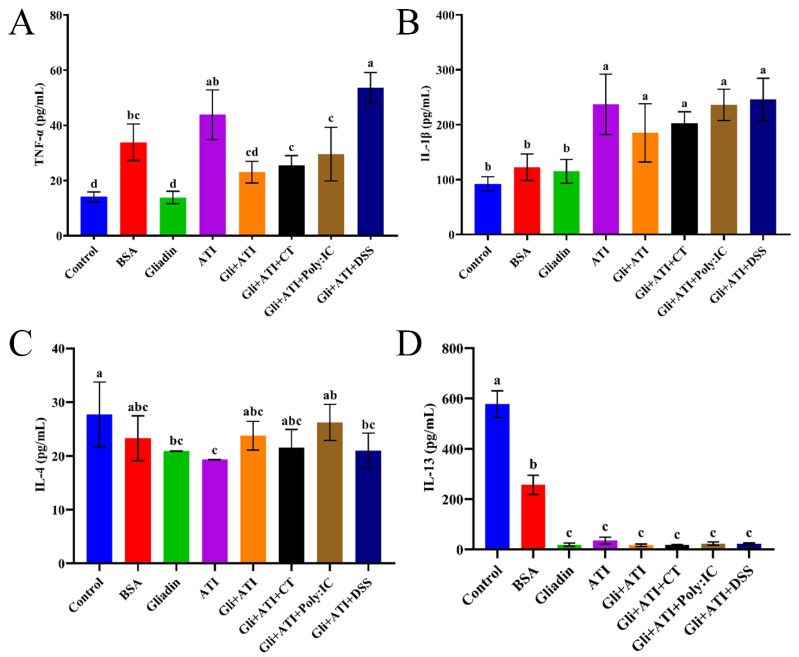
The level of (**A**) Tumor necrosis factor-α (TNF-α), (**B**) Interleukin-1β (IL-1β), (**C**) Interleukin-4 (IL-4) and (**D**) Interleukin-13 (IL-13) cytokines in spleen cell cultures of mouse. Different letters represent significant differences (*p* < 0.05) between groups.

The changes in the level of cytokines released by mouse splenocytes are shown in Figure 6. The addition of ATI increased the level of TNF-α in mice splenocytes of the Gliadin group. The level of IL-4 expressed in mice splenocytes was consistent with the flow cytometry results. Moreover, the secretion of Th1 cytokines (IL-1β) of mice whose diet had additional ATI were significantly increased (*p* < 0.05), while Th2 cytokines (IL-13) were significantly inhibited (*p* < 0.05). These noticeable differences were only reflected by comparing the experimental and control groups. Together, it had been verified again that the diet of mouse being added with Gliadin and ATI could disrupt the Th1/Th2 balance of the body and develop the direction towards the immunity of Th1.

## 4. Discussion

Animal models could mimic sensitization and intestinal inflammation of the body, which are appropriate when assessing food allergens [27,28]. The immune system of BALB/c mice is structurally analogous to that of humans, and prolonged oral challenge could trigger the immune system to counter act multiple mechanisms by stimulating innate and adaptive immune responses [29,30]. We aimed to assess the effect of wheat protein on dietary intervention of BALB/c mice by utilizing critical indicators of celiac disease-related clinical signs and pathogenesis, and ultimately to demonstrate that ATI (and related adjuvants) could aggravate the toxic effect of gliadin.

The induction of celiac disease was directly related to gliadin, and innate immune regulation played an essential role in the occurrence of the disease, which would aggravate the disease while other complications exist [31,32]. With the discovery of the ATI component in the daily diet, it had been determined that the component would seriously interfere with the immune system of gluten intolerant individuals and worsen chronic diseases [22,33]. The initial activation of TLR4 receptors by ATI in gut could trigger innate immune cells [34], which would further mobilize adaptive immune cells (T cells) that had been activated in other cells [35]. This means that ATI could activate the interaction of the innate and adaptive immune systems through TLR4 receptor in the condition of existing chronic diseases [36]. However, the direct association between ATI and celiac disease has not been reported. In our study, it could be obviously observed that the clinical symptoms of mice increased after adding ATI to the diet. Although there were no clinical reports on the extraintestinal manifestations of body temperature in patients with celiac disease, our data showed that the separate effects of ATI and gliadin caused mice to exhibit an allergy-like decrease in body temperature. The combined effect of ATI and gliadin directly increased the body temperature of mice, and we speculated that the co-action of both exacerbated the intolerance of the immune system of mice. Subsequently, we found that the combined effect of ATI and gliadin exacerbated villi atrophy, crypt hyperplasia and inflammatory cell infiltration in the intestine of mice, and it became more serious after the intervention of Poly:IC.

Celiac disease is a chronic intestinal inflammatory disease, which ultimately leads to the overexpression of tissue transglutaminase and small intestinal epithelial cell damage through the upregulation of Th1 cell subsets secreting relevant inflammatory factors [37]. The tissue transglutaminase in intestinal tissue had been identified as the characteristic indicator for screening celiac disease that was thought to perform at least two critical roles in celiac disease: serving as a deamidase which could potentiate the immunostimulatory actions of gluten, and performing as a target autoantigen in the immune response [38,39]. The expression of transglutaminase increased significantly after giving ATI to the Gliadin diet, and its expression increased further after adding Poly:IC. The expression of IL-15 in the intestines, as another characteristic indicator for identifying celiac disease, played a crucial role in the potential innate immune response of intestinal mucosa in celiac patients [40]. Consistent with tTG results, the expression level of IL-15 in mouse jejunum was further increased by the co-stimulation of ATI and Gliadin, while the additional action of Poly:IC did not further increase the expression of IL-15. Similar to previous research results [21], ATI could be used as dietary adjuvants to enhance the inflammatory response of inflammatory bowel disease or allergic diseases. We speculated that ATI might play the same role in enhancing the immunostimulatory effects of gluten proteins.

The functions of Th1 and Th2 cells were in a state of dynamic homeostasis, maintaining the normal immune response of the body. When the Th2 cells were overexpressed, the body produced allergic reactions. On the contrary, when the body regulated the development of Th2 to Th1 immune response, this would cause the body to reach an intolerant state of autoimmunity [41]. We could conclude from the T lymphocyte subsets of mouse splenocytes and related inflammatory factors that the addition of ATI to the Gliadin diet could lead to the destruction of Th1/Th2 lymphocyte subsets balance in mice, the increase in Th1 secretion of cytokines and the reduction in Th2 secretion of cytokines, eventually developing towards Th1 immunity. Our study demonstrated that the ATI which were always naturally present in gluten containing foods and in gluten preparations could exacerbate the toxic effects of gluten-induced celiac disease. It was worth noting that the differences in Th1/Th2 cell subsets and cytokine levels were only represented in the spleen for this study, and subsequent studies could focus more on intestinal immunity.

In summary, co-stimulation with wheat amylase trypsin inhibitors and gliadin could exacerbate the clinical symptoms and temperature associated with celiac disease in BALB/c mice, aggravating the intestinal villi atrophy, crypt hyperplasia and inflammatory cell infiltration of mice, as well as overexpressing tTG and IL-15 in intestinal tissues and shifting the balance of Th1/Th2 lymphocyte subsets toward Th1 immunity in the spleen. Moreover, the additional intervention of Poly:IC could aggravate intestinal damage and increase the expression of tTG in the intestine of mice. Our results systematically expounded the effects of ATI and adjuvants on the Gliadin diet in mice from the perspective of intestinal inflammation. It not only demonstrated the role of wheat amylase trypsin inhibitors in the pathogenesis of celiac disease, but also provided new ideas on the definition of a traditional gluten-free diet.

## Data Availability

Data is contained within the article or supplementary material.

## Supplementary Materials

The following supporting information can be downloaded at: https://www.mdpi.com/article/10.3390/foods11111559/s1, Figure S1: Histological grading of the mice intestine.

## References

[1] Hazard B., Trafford K., Lovegrove A., Griffiths S., Uauy C., Shewry P. (2020). Strategies to improve wheat for human health. Nat. Food.

[2] De Sousa T., Ribeiro M., Sabença C., Igrejas G. (2021). The 10,000-Year Success Story of Wheat!. Foods.

[3] Shewry P.R. (2009). Wheat. J. Exp. Bot..

[4] Sabença C., Ribeiro M., Sousa T.D., Poeta P., Bagulho A.S., Igrejas G. (2021). Wheat/Gluten-Related Disorders and Gluten-Free Diet Misconceptions: A Review. Foods.

[5] Sander I., Rihs H.-P., Doekes G., Quirce S., Krop E., Rozynek P., van Kampen V., Merget R., Meurer U., Brüning T. (2015). Component-resolved diagnosis of baker’s allergy based on specific IgE to recombinant wheat flour proteins. J. Allergy Clin. Immunol..

[6] Pronin D., Börner A., Anne Scherf K. (2020). Old and modern wheat (*Triticum aestivum* L.) cultivars and their potential to elicit celiac disease. Food Chem..

[7] Lu J., Wu Y., Yuan J., Yuan J., Wang Z., Gao J., Chen H. (2021). Characterization of AFA01 Capable of Degrading Gluten and Celiac-Immunotoxic Peptides. Foods.

[8] Martucciello S., Sposito S., Esposito C., Paolella G., Caputo I. (2020). Interplay between Type 2 Transglutaminase (TG2), Gliadin Peptide 31-43 and Anti-TG2 Antibodies in Celiac Disease. Int. J. Mol. Sci..

[9] Zhou C., Gao F., Gao J., Yuan J., Lu J., Sun Z., Xu M., Engel J., Hui W., Gilissen L. (2020). Prevalence of coeliac disease in Northwest China: Heterogeneity across Northern Silk road ethnic populations. Aliment. Pharm..

[10] Iversen R., Amundsen S.F., Kleppa L., du Pré M.F., Stamnaes J., Sollid L.M. (2020). Evidence That Pathogenic Transglutaminase 2 in Celiac Disease Derives from Enterocytes. Gastroenterology.

[11] Harris L.A., Park J.Y., Voltaggio L., Lam-Himlin D. (2012). Celiac disease: Clinical, endoscopic, and histopathologic review. Gastrointest. Endosc..

[12] Abadie V., Kim S.M., Lejeune T., Palanski B.A., Ernest J.D., Tastet O., Voisine J., Discepolo V., Marietta E.V., Hawash M.B.F. (2020). IL-15, gluten and HLA-DQ8 drive tissue destruction in coeliac disease. Nature.

[13] Junker Y., Zeissig S., Kim S.J., Barisani D., Wieser H., Leffler D.A., Zevallos V., Libermann T.A., Dillon S., Freitag T.L. (2012). Wheat amylase trypsin inhibitors drive intestinal inflammation via activation of toll-like receptor 4. J. Exp. Med..

[14] Geisslitz S., Ludwig C., Scherf K.A., Koehler P. (2018). Targeted LC-MS/MS Reveals Similar Contents of α-Amylase/Trypsin-Inhibitors as Putative Triggers of Nonceliac Gluten Sensitivity in all Wheat Species except Einkorn. J. Agric. Food Chem..

[15] Zevallos V.F., Raker V.K., Maxeiner J., Scholtes P., Steinbrink K., Schuppan D. (2019). Dietary wheat amylase trypsin inhibitors exacerbate murine allergic airway inflammation. Eur. J. Nutr..

[16] Bellinghausen I., Weigmann B., Zevallos V., Maxeiner J., Reissig S., Waisman A., Schuppan D., Saloga J. (2019). Wheat amylase-trypsin inhibitors exacerbate intestinal and airway allergic immune responses in humanized mice. J. Allergy Clin. Immunol..

[17] Tundo S., Lupi R., Lafond M., Giardina T., Larre C., Denery-Papini S., Morisset M., Kalunke R., Sestili F., Masci S. (2018). Wheat ATI CM3, CM16 and 0.28 Allergens Produced in Pichia Pastoris Display a Different Eliciting Potential in Food Allergy to Wheat (double dagger). Plants.

[18] Neumann J., Ziegler K., Gelleri M., Frohlich-Nowoisky J., Liu F., Bellinghausen I., Schuppan D., Birk U., Poschl U., Cremer C. (2019). Nanoscale distribution of TLR4 on primary human macrophages stimulated with LPS and ATI. Nanoscale.

[19] Shewry P. (2019). What Is Gluten—Why Is It Special?. Front. Nutr..

[20] Zevallos V.F., Raker V., Tenzer S., Jimenez-Calvente C., Ashfaq-Khan M., Russel N., Pickert G., Schild H., Steinbrink K., Schuppan D. (2017). Nutritional Wheat Amylase-Trypsin Inhibitors Promote Intestinal Inflammation via Activation of Myeloid Cells. Gastroenterology.

[21] Caminero A., McCarville J.L., Zevallos V.F., Pigrau M., Yu X.B., Jury J., Galipeau H.J., Clarizio A.V., Casqueiro J., Murray J.A. (2019). *Lactobacilli* Degrade Wheat Amylase Trypsin Inhibitors to Reduce Intestinal Dysfunction Induced by Immunogenic Wheat Proteins. Gastroenterology.

[22] Huang X., Schuppan D., Rojas Tovar L.E., Zevallos V.F., Loponen J., Gänzle M. (2020). Sourdough Fermentation Degrades Wheat Alpha-Amylase/Trypsin Inhibitor (ATI) and Reduces Pro-Inflammatory Activity. Foods.

[23] Tilg H., Koch R., Moschen A.R. (2013). Proinflammatory wheat attacks on the intestine: Alpha-amylase trypsin inhibitors as new players. Gastroenterology.

[24] Bai J., Hui J., Lu Q., Yang A., Yuan J., Gao J., Wu Z., Li X., Tong P., Chen H. (2020). Effect of transglutaminase cross-linking on the allergenicity of tofu based on a BALB/c mouse model. Food Funct..

[25] Vijaykrishnaraj M., Mohan Kumar B.V., Muthukumar S.P., Kurrey N.K., Prabhasankar P. (2017). Antigen-Specific Gut Inflammation and Systemic Immune Responses Induced by Prolonging Wheat Gluten Sensitization in BALB/c Murine Model. J. Proteome Res..

[26] Wickham M., Faulks R., Mills C. (2009). In vitro digestion methods for assessing the effect of food structure on allergen breakdown. Mol. Nutr. Food Res..

[27] Reyes-Pavón D., Cervantes-García D., Bermúdez-Humarán L.G., Córdova-Dávalos L.E., Quintanar-Stephano A., Jiménez M., Salinas E. (2020). Protective Effect of Glycomacropeptide on Food Allergy with Gastrointestinal Manifestations in a Rat Model through Down-Regulation of Type 2 Immune Response. Nutrients.

[28] Ladics G.S., Fry J., Goodman R., Herouet-Guicheney C., Hoffmann-Sommergruber K., Madsen C.B., Penninks A., Pomés A., Roggen E.L., Smit J. (2014). Allergic sensitization: Screening methods. Clin. Transl. Allergy.

[29] Liu T., Navarro S., Lopata A.L. (2016). Current advances of murine models for food allergy. Mol. Immunol..

[30] Oyoshi M.K., Oettgen H.C., Chatila T.A., Geha R.S., Bryce P.J. (2014). Food allergy: Insights into etiology, prevention, and treatment provided by murine models. J. Allergy Clin. Immunol..

[31] Lundin K.E.A., Wijmenga C. (2015). Coeliac disease and autoimmune disease-genetic overlap and screening. Nat. Rev. Gastroenterol. Hepatol..

[32] Leffler D.A., Green P.H.R., Fasano A. (2015). Extraintestinal manifestations of coeliac disease. Nat. Rev. Gastroenterol. Hepatol..

[33] Dos Santos Guilherme M., Zevallos V.F., Pesi A., Stoye N.M., Nguyen V.T.T., Radyushkin K., Schwiertz A., Schmitt U., Schuppan D., Endres K. (2020). Dietary Wheat Amylase Trypsin Inhibitors Impact Alzheimer’s Disease Pathology in 5xFAD Model Mice. Int. J. Mol. Sci..

[34] Bose U., Juhász A., Broadbent J.A., Byrne K., Howitt C.A., Colgrave M.L. (2020). Identification and Quantitation of Amylase Trypsin Inhibitors Across Cultivars Representing the Diversity of Bread Wheat. J. Proteome Res..

[35] Pickert G., Wirtz S., Matzner J., Ashfaq-Khan M., Heck R., Rosigkeit S., Thies D., Surabattula R., Ehmann D., Wehkamp J. (2020). Wheat Consumption Aggravates Colitis in Mice via Amylase Trypsin Inhibitor-mediated Dysbiosis. Gastroenterology.

[36] Ziegler K., Neumann J., Liu F., Fröhlich-Nowoisky J., Cremer C., Saloga J., Reinmuth-Selzle K., Pöschl U., Schuppan D., Bellinghausen I. (2018). Nitration of Wheat Amylase Trypsin Inhibitors Increases Their Innate and Adaptive Immunostimulatory Potential. Front. Immunol..

[37] Lebwohl B., Sanders D.S., Green P.H.R. (2018). Coeliac disease. Lancet.

[38] Di Sabatino A., Vanoli A., Giuffrida P., Luinetti O., Solcia E., Corazza G.R. (2012). The function of tissue transglutaminase in celiac disease. Autoimmun. Rev..

[39] Freitag T., Schulze-Koops H., Niedobitek G., Melino G., Schuppan D. (2004). The role of the immune response against tissue transglutaminase in the pathogenesis of coeliac disease. Autoimmun. Rev..

[40] Abadie V., Jabri B. (2014). IL-15: A central regulator of celiac disease immunopathology. Immunol. Rev..

[41] Annunziato F., Romagnani C., Romagnani S. (2015). The 3 major types of innate and adaptive cell-mediated effector immunity. J. Allergy Clin. Immunol..

