# Supplementary material for: Wheat Amylase Trypsin Inhibitors Aggravate Intestinal Inflammation Associated with Celiac Disease Mediated by Gliadin in BALB/c Mice
Source: Foods. 2022 May 25;11(11):1559. doi: 10.3390/foods11111559 (PMC9180791; doi:10.3390/foods11111559)
Supplement: Supplementary file 1 [file foods-11-01559-s001.zip › foods-1670231-supplementary.pdf]

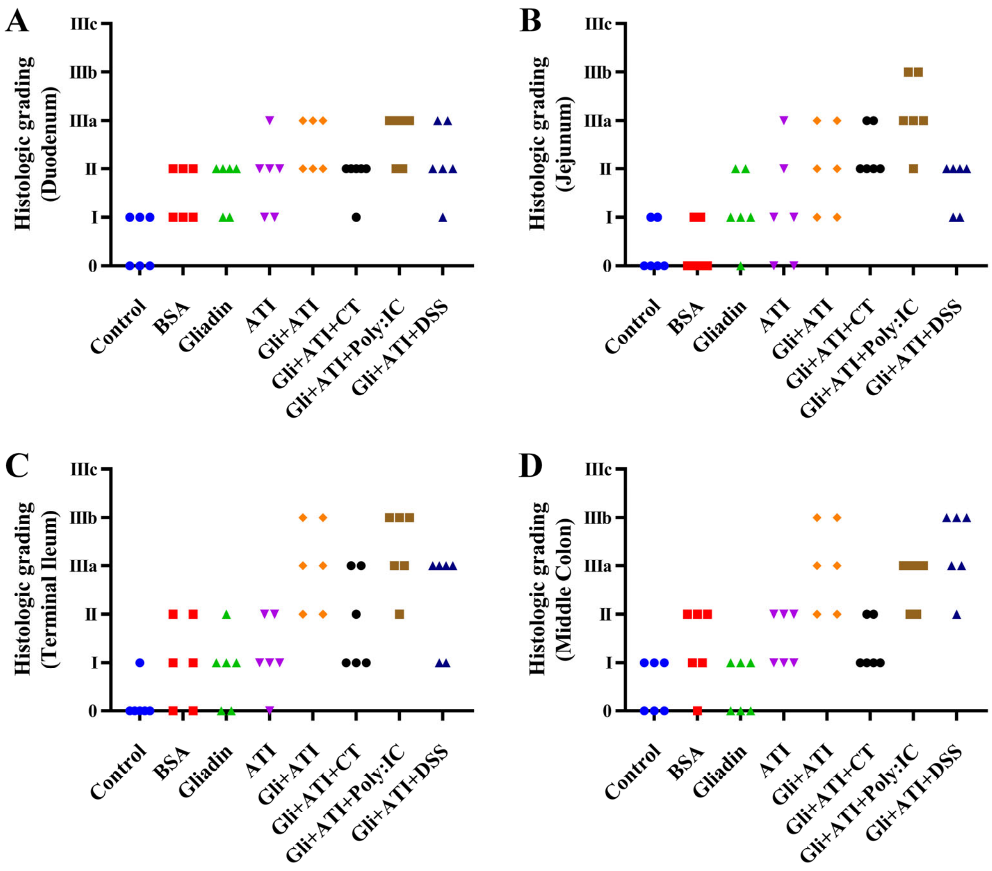

Supplementary Figure S1. Histological grading of the mice intestine. (A) Duodenum. (B) Jejunum. (C) Terminal Ileum. (D) Middle Colon.

Footnote: Marsh–Oberhuber histologic grading in celiac disease: (0) Normal—normal-appearing villus architecture; (I) Infiltrative—normal mucosal and villus architecture; increased numbers of intraepithelial lymphocytes (IEL); (II) Hyperplastic—similar to 1 with enlarged crypts and increased crypt cell division; (IIIa) Partial villus atrophy—shortened, blunt villi; mild lymphocyte infiltration, enlarged hyperplastic crypts; (IIIb) Subtotal villus atrophy—clearly atrophic villi, enlarged crypts whose immature epithelial cells are generated at an increased rate, influx of inflammatory cells; (IIIc) Hypoplastic—total villus atrophy, complete loss of villi, severe crypt hyperplasia, infiltrative inflammatory lesion.
